# Supplementary material for: A Systematic Computational Framework for Practical Identifiability Analysis in Mathematical Models Arising from Biology
Source: Adv Sci (Weinh). 2025 Jul 22;12(35):e04346. doi: 10.1002/advs.202504346 (PMC12463036; doi:10.1002/advs.202504346)
Supplement: Supplementary file 1 — Supporting Information [file ADVS-12-e04346-s001.pdf]

ADVANCED SCIENCE
Open Access

Supporting Information

for *Adv. Sci.*, DOI 10.1002/advs.202504346

A Systematic Computational Framework for Practical Identifiability Analysis in Mathematical Models Arising from Biology

*Shun Wang and Wenrui Hao**

A Systematic Computational Framework for Practical Identifiability Analysis in Mathematical Models Arising from Biology

Shun Wang[1], Wenrui Hao[1,*]

Content

Section 1 Theoretical Analysis 1

Proof of Theorem 1 1

Proof of Theorem 2 4

Proof of Theorem 3 5

Proof of Theorem 4 6

Section 2 Supplementary Figures 6

Section 3 Applications of Practical Identifiability 12

Section 4 Values of the parameters.. 16

Section 5 Supplementary Tables 18

References.. 20

Section 1 Theoretical Analysis

Proof of Theorem 1

Proof: Before starting the proof, we set $L = 1$ for the convenience of the subsequent proof. The elements of the FIM are defined as

$$F(\boldsymbol{\theta}) = \left\{F(\boldsymbol{\theta})_{ij}\right\}_{i,j=1,2,\ldots,k} = E\left(\frac{\partial \log \mathcal{L}(\widehat{\boldsymbol{h}};\boldsymbol{\theta})}{\partial \theta_i}\frac{\partial \log \mathcal{L}(\widehat{\boldsymbol{h}};\boldsymbol{\theta})}{\partial \theta_j}\right), \qquad (S1)$$

Where $\mathcal{L}(\widehat{\boldsymbol{h}};\boldsymbol{\theta})$ is the likelihood function $\left(\mathcal{L}(\widehat{\boldsymbol{h}};\boldsymbol{\theta}) > 0, \int \mathcal{L}(\widehat{\boldsymbol{h}};\boldsymbol{\theta})d\widehat{\boldsymbol{h}} = 1\right)$. We assume that $F(\boldsymbol{\theta})$ exist and are continuous functions of $\boldsymbol{\theta}$ in the parameter space Θ (1).

Assuming that the experiment measurements are contaminated by additive

normally distributed uncorrelated random measurement errors (2), i.e. $\hat{h}_i = h(\boldsymbol{\varphi}(t_i,\boldsymbol{\theta})) + \varepsilon_i, \varepsilon_i \sim \mathcal{N}(0,\sigma^2)$, the estimation of the model parameters is formulated as the maximization of the likelihood of the data as follows:

$$\mathcal{L}(\hat{\boldsymbol{h}};\boldsymbol{\theta}) = \frac{1}{((2\pi)^N|\Sigma|)^{1/2}}\exp\left(-\frac{1}{2}\left(\hat{\boldsymbol{h}} - \boldsymbol{h}(\boldsymbol{\varphi}(\boldsymbol{t},\boldsymbol{\theta}))\right)^T \Sigma^{-1}\left(\hat{\boldsymbol{h}} - \boldsymbol{h}(\boldsymbol{\varphi}(\boldsymbol{t},\boldsymbol{\theta}))\right)\right), (S2)$$

where the vector $\boldsymbol{h}(\boldsymbol{\varphi}(\boldsymbol{t},\boldsymbol{\theta})) = \left(h(\boldsymbol{\varphi}(t_1,\boldsymbol{\theta})), h(\boldsymbol{\varphi}(t_2,\boldsymbol{\theta})), \dots, h(\boldsymbol{\varphi}(t_N,\boldsymbol{\theta}))\right)^T$ is the system output with parameter $\boldsymbol{\theta}$ and the experiment data is denoted as $\hat{\boldsymbol{h}} = (\hat{h}_1, \hat{h}_2, \dots, \hat{h}_N)^T$. The covariance matrix $\Sigma = \sigma^2 I$, and I is the identity matrix. The logarithmic likelihood function $l(\hat{\boldsymbol{h}};\boldsymbol{\theta}) \equiv -\log\mathcal{L}(\hat{\boldsymbol{h}};\boldsymbol{\theta})$ is given as

$$l(\hat{\boldsymbol{h}};\boldsymbol{\theta}) = \frac{1}{2\sigma^2}\left(\hat{\boldsymbol{h}} - \boldsymbol{h}(\boldsymbol{\varphi}(\boldsymbol{t},\boldsymbol{\theta}))\right)^T\left(\hat{\boldsymbol{h}} - \boldsymbol{h}(\boldsymbol{\varphi}(\boldsymbol{t},\boldsymbol{\theta}))\right) + \frac{N}{2}\log 2\pi + \frac{1}{2}\log\sigma^2. (S3)$$

For the small perturbation $(\boldsymbol{\delta})$ in experimental data, the logarithmic likelihood function $l(\hat{\boldsymbol{h}} - \boldsymbol{\delta};\boldsymbol{\theta})$ is changed as:

$$l(\hat{\boldsymbol{h}} - \boldsymbol{\delta};\boldsymbol{\theta}) = \frac{1}{2\sigma^2}\left(\hat{\boldsymbol{h}} - \boldsymbol{h}(\boldsymbol{\varphi}(\boldsymbol{t},\boldsymbol{\theta}))\right)^T\left(\hat{\boldsymbol{h}} - \boldsymbol{h}(\boldsymbol{\varphi}(\boldsymbol{t},\boldsymbol{\theta}))\right)$$
$$-\frac{1}{\sigma^2}\boldsymbol{\delta}^T\left(\hat{\boldsymbol{h}} - \boldsymbol{h}(\boldsymbol{\varphi}(\boldsymbol{t},\boldsymbol{\theta}))\right) + \frac{1}{2\sigma^2}\boldsymbol{\delta}^T\boldsymbol{\delta} + \frac{N}{2}\log 2\pi + \frac{1}{2}\log\sigma^2. \quad (S4)$$

The relative entropy is utilized to measure the distribution distance between $\mathcal{L}(\hat{\boldsymbol{h}};\boldsymbol{\theta}^*)$ and $\mathcal{L}(\hat{\boldsymbol{h}} - \boldsymbol{\delta};\boldsymbol{\theta}_\delta)$ as

$$D\left(\mathcal{L}(\hat{\boldsymbol{h}};\boldsymbol{\theta}^*) : \mathcal{L}(\hat{\boldsymbol{h}} - \boldsymbol{\delta};\boldsymbol{\theta}_\delta)\right) = \int \mathcal{L}(\hat{\boldsymbol{h}};\boldsymbol{\theta}^*)\log\frac{\mathcal{L}(\hat{\boldsymbol{h}};\boldsymbol{\theta}^*)}{\mathcal{L}(\hat{\boldsymbol{h}} - \boldsymbol{\delta};\boldsymbol{\theta}_\delta)}d\hat{\boldsymbol{h}}, (S5)$$

Using the Taylor expansion to second order, we have

$$D\left(\mathcal{L}(\hat{\boldsymbol{h}};\boldsymbol{\theta}^*) : \mathcal{L}(\hat{\boldsymbol{h}} - \boldsymbol{\delta};\boldsymbol{\theta}_\delta)\right) = \int \mathcal{L}(\hat{\boldsymbol{h}};\boldsymbol{\theta}^*)\,(\log\mathcal{L}(\hat{\boldsymbol{h}};\boldsymbol{\theta}^*)$$
$$-\log\mathcal{L}(\hat{\boldsymbol{h}} - \boldsymbol{\delta};\boldsymbol{\theta}^*) - \nabla_{\boldsymbol{\theta}}\log\mathcal{L}(\hat{\boldsymbol{h}} - \boldsymbol{\delta};\boldsymbol{\theta}^*)^T(\boldsymbol{\theta}_\delta - \boldsymbol{\theta}^*)$$
$$-\frac{1}{2}(\boldsymbol{\theta}_\delta - \boldsymbol{\theta}^*)^T H\left(\log\mathcal{L}(\hat{\boldsymbol{h}} - \boldsymbol{\delta};\boldsymbol{\theta}^*)\right)(\boldsymbol{\theta}_\delta - \boldsymbol{\theta}^*) + o(\|\boldsymbol{\theta}_\delta - \boldsymbol{\theta}^*\|^2)\boldsymbol{d}\hat{\boldsymbol{h}}. (S6)$$

where $H\left(\log\mathcal{L}(\hat{\boldsymbol{h}} - \boldsymbol{\delta};\boldsymbol{\theta}^*)\right)$ is the Hessian matrix of $l(\hat{\boldsymbol{h}} - \boldsymbol{\delta};\boldsymbol{\theta}^*)$. Hence, these formulars can be given as

$$\int \mathcal{L}(\hat{\boldsymbol{h}};\boldsymbol{\theta}^*)\left(\log\mathcal{L}(\hat{\boldsymbol{h}};\boldsymbol{\theta}^*) - \log\mathcal{L}(\hat{\boldsymbol{h}} - \boldsymbol{\delta};\boldsymbol{\theta}^*)\right)\boldsymbol{d}\hat{\boldsymbol{h}}$$
$$= E\left[-\frac{1}{\sigma^2}\boldsymbol{\delta}^T\left(\hat{\boldsymbol{h}} - \boldsymbol{h}(\boldsymbol{\varphi}(\boldsymbol{t},\boldsymbol{\theta}^*))\right) + \frac{1}{2\sigma^2}\boldsymbol{\delta}^T\boldsymbol{\delta}\right] = \frac{1}{2\sigma^2}\boldsymbol{\delta}^T\boldsymbol{\delta}. (10)$$

$$\frac{\partial l(\widehat{\boldsymbol{h}}-\boldsymbol{\delta};\boldsymbol{\theta})}{\partial\theta_i}=\frac{1}{\sigma^2}\left(\frac{\partial \boldsymbol{h}(\boldsymbol{\varphi}(\boldsymbol{t},\boldsymbol{\theta}^*))}{\partial\theta_i}\right)^T\left(\widehat{\boldsymbol{h}}-\boldsymbol{h}(\boldsymbol{\varphi}(\boldsymbol{t},\boldsymbol{\theta}))\right)-\frac{1}{\sigma^2}\boldsymbol{\delta}^T\frac{\partial \boldsymbol{h}(\boldsymbol{\varphi}(\boldsymbol{t},\boldsymbol{\theta}^*))}{\partial\theta_i},\quad (S7)$$

$$\int \mathcal{L}(\widehat{\boldsymbol{h}};\boldsymbol{\theta}^*)\left(-\nabla_{\boldsymbol{\theta}}\log\mathcal{L}(\widehat{\boldsymbol{h}}-\boldsymbol{\delta};\boldsymbol{\theta}^*)^T\right)d\widehat{\boldsymbol{h}}$$

$$=E\left[\frac{1}{\sigma^2}\left(\widehat{\boldsymbol{h}}-\boldsymbol{h}(\boldsymbol{\varphi}(\boldsymbol{t},\boldsymbol{\theta}^*))\right)^T\nabla_{\boldsymbol{\theta}}\boldsymbol{h}(\boldsymbol{\varphi}(\boldsymbol{t},\boldsymbol{\theta}^*))^T-\frac{1}{\sigma^2}\boldsymbol{\delta}^T\nabla_{\boldsymbol{\theta}}\boldsymbol{h}(\boldsymbol{\varphi}(\boldsymbol{t},\boldsymbol{\theta}^*))^T\right]$$

$$=-\frac{1}{\sigma^2}\boldsymbol{\delta}^T\nabla_{\theta}\boldsymbol{h}(\boldsymbol{\varphi}(\boldsymbol{t},\boldsymbol{\theta}^*))^T\ (S8)$$

The FIM $F(\boldsymbol{\theta})=\{F(\boldsymbol{\theta})_{ij}\}_{i,j=1,2,\ldots,k}$ at the parameter $\boldsymbol{\theta}^*$ is obtained as follows:

$$\frac{\partial l(\widehat{\boldsymbol{h}}-\boldsymbol{\delta};\boldsymbol{\theta})}{\partial\theta_i}=\frac{1}{\sigma^2}\left(\frac{\partial \boldsymbol{h}(\boldsymbol{\varphi}(\boldsymbol{t},\boldsymbol{\theta}))}{\partial\theta_i}\right)^T\left(\widehat{\boldsymbol{h}}-\boldsymbol{h}(\boldsymbol{\varphi}(\boldsymbol{t},\boldsymbol{\theta}))\right)-\frac{1}{\sigma^2}\boldsymbol{\delta}^T\frac{\partial \boldsymbol{h}(\boldsymbol{\varphi}(\boldsymbol{t},\boldsymbol{\theta}))}{\partial\theta_i},\quad (S9)$$

$$\frac{\partial l(\widehat{\boldsymbol{h}}-\boldsymbol{\delta};\boldsymbol{\theta})}{\partial\theta_j}=\frac{1}{\sigma^2}\left(\frac{\partial \boldsymbol{h}(\boldsymbol{\varphi}(\boldsymbol{t},\boldsymbol{\theta}))}{\partial\theta_j}\right)^T\left(\widehat{\boldsymbol{h}}-\boldsymbol{h}(\boldsymbol{\varphi}(\boldsymbol{t},\boldsymbol{\theta}))\right)-\frac{1}{\sigma^2}\boldsymbol{\delta}^T\frac{\partial \boldsymbol{h}(\boldsymbol{\varphi}(t,\boldsymbol{\theta}))}{\partial\theta_j},\quad (S10)$$

$$E\left(\frac{\partial l(\widehat{\boldsymbol{h}}-\boldsymbol{\delta};\boldsymbol{\theta}^*)}{\partial\theta_i}\frac{\partial l(\widehat{\boldsymbol{h}}-\boldsymbol{\delta};\boldsymbol{\theta}^*)}{\partial\theta_j}\right)$$

$$=\frac{1}{\sigma^4}E\left(\left(\frac{\partial \boldsymbol{h}(\boldsymbol{\varphi}(\boldsymbol{t},\boldsymbol{\theta}^*))}{\partial\theta_i}\right)^T\left(\widehat{\boldsymbol{h}}-\boldsymbol{h}(\boldsymbol{\varphi}(\boldsymbol{t},\boldsymbol{\theta}^*))\right)\left(\frac{\partial \boldsymbol{h}(\boldsymbol{\varphi}(\boldsymbol{t},\boldsymbol{\theta}^*))}{\partial\theta_j}\right)^T\left(\widehat{\boldsymbol{h}}-\boldsymbol{h}(\boldsymbol{\varphi}(\boldsymbol{t},\boldsymbol{\theta}^*))\right)\right)$$

$$-\frac{2}{\sigma^4}E\left(\boldsymbol{\delta}^T\frac{\partial \boldsymbol{h}(\boldsymbol{\varphi}(\boldsymbol{t},\boldsymbol{\theta}^*))}{\partial\theta_i}\left(\frac{\partial \boldsymbol{h}(\boldsymbol{\varphi}(\boldsymbol{t},\boldsymbol{\theta}^*))}{\partial\theta_j}\right)^T\left(\widehat{\boldsymbol{h}}-\boldsymbol{h}(\boldsymbol{\varphi}(\boldsymbol{t},\boldsymbol{\theta}^*))\right)\right)$$

$$+\frac{1}{\sigma^4}\boldsymbol{\delta}^T\frac{\partial \boldsymbol{h}(\boldsymbol{\varphi}(\boldsymbol{t},\boldsymbol{\theta}^*))}{\partial\theta_i}\left(\frac{\partial \boldsymbol{h}(\boldsymbol{\varphi}(\boldsymbol{t},\boldsymbol{\theta}^*))}{\partial\theta_j}\right)^T\boldsymbol{\delta}$$

$$=\frac{1}{\sigma^4}E\left(\left(\frac{\partial \boldsymbol{h}(\boldsymbol{\varphi}(\boldsymbol{t},\boldsymbol{\theta}^*))}{\partial\theta_i}\right)^T\left(\widehat{\boldsymbol{h}}-\boldsymbol{h}(\boldsymbol{\varphi}(\boldsymbol{t},\boldsymbol{\theta}^*))\right)\left(\frac{\partial \boldsymbol{h}(\boldsymbol{\varphi}(\boldsymbol{t},\boldsymbol{\theta}^*))}{\partial\theta_j}\right)^T\left(\widehat{\boldsymbol{h}}-\boldsymbol{h}(\boldsymbol{\varphi}(\boldsymbol{t},\boldsymbol{\theta}^*))\right)\right)$$

$$+\frac{1}{\sigma^4}\left(\frac{\partial \boldsymbol{h}(\boldsymbol{\varphi}(\boldsymbol{t},\boldsymbol{\theta}^*))}{\partial\theta_i}\right)^T\boldsymbol{\delta}\boldsymbol{\delta}^T\frac{\partial \boldsymbol{h}(\boldsymbol{\varphi}(\boldsymbol{t},\boldsymbol{\theta}^*))}{\partial\theta_j}.$$

$$\left(E\left(\left(\widehat{\boldsymbol{h}}-\boldsymbol{h}(\boldsymbol{\varphi}(\boldsymbol{t},\boldsymbol{\theta}^*))\right)\left(\widehat{\boldsymbol{h}}-\boldsymbol{h}(\boldsymbol{\varphi}(\boldsymbol{t},\boldsymbol{\theta}^*))\right)^T\right)=\Sigma\right)$$

$$=\frac{1}{\sigma^2}\left(\frac{\partial \boldsymbol{h}(\boldsymbol{\varphi}(\boldsymbol{t},\boldsymbol{\theta}^*))}{\partial\theta_i}\right)^T\frac{\partial \boldsymbol{h}(\boldsymbol{\varphi}(\boldsymbol{t},\boldsymbol{\theta}^*))}{\partial\theta_j}+\frac{1}{\sigma^4}\left(\frac{\partial \boldsymbol{h}(\boldsymbol{\varphi}(\boldsymbol{t},\boldsymbol{\theta}^*))}{\partial\theta_i}\right)^T\boldsymbol{\delta}\boldsymbol{\delta}^T\frac{\partial \boldsymbol{h}(\boldsymbol{\varphi}(\boldsymbol{t},\boldsymbol{\theta}^*))}{\partial\theta_j}\ (S11)$$

Hence, we have

$$\int \mathcal{L}(\widehat{\boldsymbol{h}};\boldsymbol{\theta})\left(-(\boldsymbol{\theta}_{\boldsymbol{\delta}}-\boldsymbol{\theta}^*)^T H_{ij}\left(\log\mathcal{L}(\widehat{\boldsymbol{h}}-\boldsymbol{\delta};\boldsymbol{\theta}^*)\right)(\boldsymbol{\theta}_{\boldsymbol{\delta}}-\boldsymbol{\theta}^*)\right)d\widehat{\boldsymbol{h}}$$

$$= (\boldsymbol{\theta_\delta} - \boldsymbol{\theta}^*)^T \left(-E\left(H_{ij}\left(\log \mathcal{L}\left(\widehat{\boldsymbol{h}} - \boldsymbol{\delta}; \boldsymbol{\theta}^*\right)\right)\right)\right)(\boldsymbol{\theta_\delta} - \boldsymbol{\theta}^*)$$

$$= (\boldsymbol{\theta_\delta} - \boldsymbol{\theta}^*)^T \frac{\partial l\left(\widehat{\boldsymbol{h}} - \boldsymbol{\delta}; \boldsymbol{\theta}^*\right)}{\partial \theta_i} \frac{\partial l\left(\widehat{\boldsymbol{h}} - \boldsymbol{\delta}; \boldsymbol{\theta}^*\right)}{\partial \theta_j} (\boldsymbol{\theta_\delta} - \boldsymbol{\theta}^*)$$

$$= \frac{1}{\sigma^2} (\boldsymbol{\theta_\delta} - \boldsymbol{\theta}^*)^T \left(\frac{\partial \boldsymbol{h}\left(\boldsymbol{\varphi}(\boldsymbol{t}, \boldsymbol{\theta}^*)\right)}{\partial \theta_i}\right)^T \left(\frac{\partial \boldsymbol{h}\left(\boldsymbol{\varphi}(\boldsymbol{t}, \boldsymbol{\theta}^*)\right)}{\partial \theta_j}\right) (\boldsymbol{\theta_\delta} - \boldsymbol{\theta}^*)$$

$$+ \frac{1}{\sigma^4} (\boldsymbol{\theta_\delta} - \boldsymbol{\theta}^*)^T \left(\frac{\partial \boldsymbol{h}\left(\boldsymbol{\varphi}(\boldsymbol{t}, \boldsymbol{\theta}^*)\right)}{\partial \theta_i}\right)^T \boldsymbol{\delta}\boldsymbol{\delta}^T \frac{\partial \boldsymbol{h}\left(\boldsymbol{\varphi}(\boldsymbol{t}, \boldsymbol{\theta}^*)\right)}{\partial \theta_j} (\boldsymbol{\theta_\delta} - \boldsymbol{\theta}^*). (S12)$$

Now, we obtain the relative entropy $D\left(\mathcal{L}\left(\widehat{\boldsymbol{h}}; \boldsymbol{\theta}^*\right) : \mathcal{L}\left(\widehat{\boldsymbol{h}} - \boldsymbol{\delta}; \boldsymbol{\theta_\delta}\right)\right)$ as:

$$D\left(\mathcal{L}\left(\widehat{\boldsymbol{h}}; \boldsymbol{\theta}^*\right) : \mathcal{L}\left(\widehat{\boldsymbol{h}} - \boldsymbol{\delta}; \boldsymbol{\theta_\delta}\right)\right) = \frac{1}{2\sigma^2} \boldsymbol{\delta}^T \boldsymbol{\delta} - \frac{1}{\sigma^2} \boldsymbol{\delta}^T \nabla_\theta \boldsymbol{h}\left(\boldsymbol{\varphi}(\boldsymbol{t}, \boldsymbol{\theta}^*)\right)^T (\boldsymbol{\theta_\delta} - \boldsymbol{\theta}^*)$$

$$+ \frac{1}{2} (\boldsymbol{\theta_\delta} - \boldsymbol{\theta}^*)^T F_{\boldsymbol{\delta}}(\boldsymbol{\theta}^*) (\boldsymbol{\theta_\delta} - \boldsymbol{\theta}^*) + o(\|\boldsymbol{\theta_\delta} - \boldsymbol{\theta}^*\|^2), (S13)$$

where $F_{\boldsymbol{\delta}}(\boldsymbol{\theta}^*) = E\left(\frac{\partial \log \mathcal{L}\left(\widehat{\boldsymbol{h}} - \boldsymbol{\delta}; \boldsymbol{\theta}^*\right)}{\partial \theta_i} \frac{\partial \log \mathcal{L}\left(\widehat{\boldsymbol{h}} - \boldsymbol{\delta}; \boldsymbol{\theta}^*\right)}{\partial \theta_j}\right)$ is the FIM at the parameter $\boldsymbol{\theta}^*$ as follows:

$$\{F_{\boldsymbol{\delta}}(\boldsymbol{\theta}^*)\}_{ij} = \{F(\boldsymbol{\theta}^*)\}_{ij} + \frac{1}{\sigma^4} \left(\frac{\partial \boldsymbol{h}\left(\boldsymbol{\varphi}(\boldsymbol{t}, \boldsymbol{\theta}^*)\right)}{\partial \theta_i}\right)^T \boldsymbol{\delta}\boldsymbol{\delta}^T \frac{\partial \boldsymbol{h}\left(\boldsymbol{\varphi}(\boldsymbol{t}, \boldsymbol{\theta}^*)\right)}{\partial \theta_j},$$

$$\{F(\boldsymbol{\theta}^*)\}_{ij} = \frac{1}{\sigma^2} \left(\frac{\partial \boldsymbol{h}\left(\boldsymbol{\varphi}(\boldsymbol{t}, \boldsymbol{\theta}^*)\right)}{\partial \theta_i}\right)^T \left(\frac{\partial \boldsymbol{h}\left(\boldsymbol{\varphi}(\boldsymbol{t}, \boldsymbol{\theta}^*)\right)}{\partial \theta_j}\right) (S14)$$

Based on the definition of practical identifiability (**Definition 1**) as

$$\lim_{\|\boldsymbol{\delta}\| \to 0} D\left(\mathcal{L}\left(\widehat{\boldsymbol{h}}; \boldsymbol{\theta}^*\right) : \mathcal{L}\left(\widehat{\boldsymbol{h}} - \boldsymbol{\delta}; \boldsymbol{\theta_\delta}\right)\right) = 0, \lim_{\|\boldsymbol{\delta}\| \to 0} \|\boldsymbol{\theta_\delta} - \boldsymbol{\theta}^*\| = 0, (S15)$$

we have

$$\lim_{\|\boldsymbol{\delta}\| \to 0} D\left(\mathcal{L}\left(\widehat{\boldsymbol{h}}; \boldsymbol{\theta}^*\right) : \mathcal{L}\left(\widehat{\boldsymbol{h}} - \boldsymbol{\delta}; \boldsymbol{\theta_\delta}\right)\right) = \lim_{\|\boldsymbol{\delta}\| \to 0} \frac{1}{2} (\boldsymbol{\theta}_\delta - \boldsymbol{\theta}^*)^T F(\boldsymbol{\theta}^*) (\boldsymbol{\theta}_\delta - \boldsymbol{\theta}^*) = 0. (S16)$$

$\lim_{\|\boldsymbol{\delta}\| \to 0} \|\boldsymbol{\theta}_\delta - \boldsymbol{\theta}^*\| = 0$ if and only if $F(\boldsymbol{\theta}^*)$ is invertible. This conclusion can be generalized to cases where $L > 1$. □

Proof of Theorem 2

Proof: On the one hand, the necessary condition for $\min_{\boldsymbol{\theta}} l\left(\widehat{\boldsymbol{h}}; \boldsymbol{\theta}\right)$ is obtained as

$$\boldsymbol{S}^T \boldsymbol{S} \boldsymbol{\theta} = \boldsymbol{S}^T \boldsymbol{b}. (S17)$$

For $\forall \theta_i^*, i = 1,2, \ldots, k$, based on the **Definition 2**, we optimize the conditional loss function as $\min_{\theta_{j,j \neq i}} l\left(\widehat{\boldsymbol{h}}; \boldsymbol{\theta} | \theta_i\right)$. First, we perform the elementary matrix

transformation to the matrix $\boldsymbol{S}$ as $\widehat{\boldsymbol{S}} = \boldsymbol{S}\boldsymbol{P}_{\boldsymbol{i},\boldsymbol{k}}$, where the matrix $\boldsymbol{P}_{\boldsymbol{i},\boldsymbol{k}} = [\boldsymbol{e}_1, \boldsymbol{e}_2, \cdots, \boldsymbol{e}_{i-1}, \boldsymbol{e}_k, \boldsymbol{e}_{i+1}, \dots, \boldsymbol{e}_{k-1}, \boldsymbol{e}_i]$ is the elementary matrix and the vector $\boldsymbol{e}_i$ is unite vector. Next, we decompose the matrix $\widehat{\boldsymbol{S}}$ as $\widehat{\boldsymbol{S}} = [\boldsymbol{A}, \boldsymbol{s}_i], \boldsymbol{A} \in \mathbb{R}^{n\times(k-1)}, \boldsymbol{s}_i \in \mathbb{R}^n$ $(n = N * L \geq k)$, $\boldsymbol{s}_i$ is the ith column of matrix $\boldsymbol{S}$. The parameter $\boldsymbol{\theta}$ is rewritten as $\boldsymbol{\theta} = [\widetilde{\boldsymbol{\theta}}; \theta_i], \widetilde{\boldsymbol{\theta}} \in \mathbb{R}^{k-1}$**.** The conditional loss function $\min_{\theta_{j,j\neq i}} l(\widehat{\boldsymbol{h}}; \boldsymbol{\theta}|\theta_i)$ is rewritten as

$$\min_{\widetilde{\boldsymbol{\theta}}} l(\widehat{\boldsymbol{h}}; \widetilde{\boldsymbol{\theta}}|\theta_i) \approx \min_{\widetilde{\boldsymbol{\theta}}} \left\|\boldsymbol{A}\widetilde{\boldsymbol{\theta}} + \boldsymbol{s}_i\theta_i - \boldsymbol{b}\right\|_2^2. (S18)$$

Using the condition that $F(\boldsymbol{\theta}^*) = \boldsymbol{S}^T\boldsymbol{S}$ is invertible, the matrix $\hat{F}(\boldsymbol{\theta}^*) = \widehat{\boldsymbol{S}}^T\widehat{\boldsymbol{S}} = \boldsymbol{P}_{\boldsymbol{i},\boldsymbol{k}} F(\boldsymbol{\theta}^*)\boldsymbol{P}_{\boldsymbol{i},\boldsymbol{k}}$ is also invertible, and the matrix $\boldsymbol{A}$ is full rank of column. Utilizing the necessary condition of least square, the parameter $\widetilde{\boldsymbol{\theta}}$ is given as:

$$\widetilde{\boldsymbol{\theta}} = \boldsymbol{A}^\dagger(\boldsymbol{b} - \boldsymbol{s}_i\theta_i),\ \ \boldsymbol{A}^\dagger = (\boldsymbol{A}^T\boldsymbol{A})^{-1}\boldsymbol{A}^T. (S19)$$

Substituted into the conditional loss function, we can obtain the equation as

$$l(\widehat{\boldsymbol{h}}; \widetilde{\boldsymbol{\theta}}|\theta_i) \approx \left\|\boldsymbol{A}\boldsymbol{A}^\dagger\boldsymbol{b} - \boldsymbol{b} + (\boldsymbol{I} - \boldsymbol{A}\boldsymbol{A}^\dagger)\boldsymbol{s}_i\theta_i\right\|_2^2. (S20)$$

Based on the **Definition 2**, the parameter $\theta_i^* \in \boldsymbol{\theta}^*$ is identifiable when $l(\widehat{\boldsymbol{h}}; \boldsymbol{\theta}|\theta_i)$ is convex function. The loss function $l(\widehat{\boldsymbol{h}}; \widetilde{\boldsymbol{\theta}}|\theta_i)$ is convex if and only if $(\boldsymbol{I} - \boldsymbol{A}\boldsymbol{A}^\dagger)\boldsymbol{s}_i \neq \boldsymbol{0}$. Because the matrix $\boldsymbol{A}$ is full rank of column, we have

$$\begin{vmatrix}(\boldsymbol{A}^T\boldsymbol{A})^{-1} & \boldsymbol{0} \\ \boldsymbol{0} & 1\end{vmatrix} \det\left(\hat{F}(\boldsymbol{\theta}^*)\right) = \begin{vmatrix}\boldsymbol{I}_{(k-1)\times(k-1)} & \boldsymbol{A}^\dagger\boldsymbol{s}_i \\ \boldsymbol{s}_i^T\boldsymbol{A} & \boldsymbol{s}_i^T\boldsymbol{s}_i\end{vmatrix} \neq 0, \boldsymbol{s}_i^T(\boldsymbol{s}_i - \boldsymbol{A}\boldsymbol{A}^\dagger\boldsymbol{s}_i) \neq 0.\ (S21)$$

Because the matrix $\boldsymbol{S}$ is full rank of column, $\boldsymbol{s}_i$ is not zero vector. So, we have $(\boldsymbol{s}_i - \boldsymbol{A}\boldsymbol{A}^\dagger\boldsymbol{s}_i) \neq \boldsymbol{0}$.

On the other hand, if $(\boldsymbol{I} - \boldsymbol{A}\boldsymbol{A}^\dagger)\boldsymbol{s}_i \neq \boldsymbol{0}$**,** the vector $\boldsymbol{s}_i$ is decomposed as:

$$\boldsymbol{s}_i = \boldsymbol{s}_i^{(1)} + \boldsymbol{s}_i^{(2)}, \qquad \boldsymbol{s}_i^{(1)} = \boldsymbol{A}\boldsymbol{A}^\dagger\boldsymbol{s}_i, \qquad \boldsymbol{s}_i^{(2)} = (\boldsymbol{I} - \boldsymbol{A}\boldsymbol{A}^\dagger)\boldsymbol{s}_i, \qquad \boldsymbol{s}_i^{(1)} \perp \boldsymbol{s}_i^{(2)}$$

$$\boldsymbol{s}_i^T(\boldsymbol{s}_i - \boldsymbol{A}\boldsymbol{A}^\dagger\boldsymbol{s}_i) = \left(\boldsymbol{s}_i^{(2)}\right)^T\boldsymbol{s}_i^{(2)} > 0, \begin{vmatrix}(\boldsymbol{A}^T\boldsymbol{A})^{-1} & \boldsymbol{0} \\ \boldsymbol{0} & 1\end{vmatrix} \det\left(\hat{F}(\boldsymbol{\theta}^*)\right) > 0. (S22)$$

So, $F(\boldsymbol{\theta}^*) = \boldsymbol{P}_{\boldsymbol{i},\boldsymbol{k}}^{-1} \hat{F}(\boldsymbol{\theta}^*)\boldsymbol{P}_{\boldsymbol{i},\boldsymbol{k}}^{-1}$ is invertible where $\boldsymbol{P}_{\boldsymbol{i},\boldsymbol{k}}$ is the elementary matrix. □

Proof of Theorem 3

Proof: If $\boldsymbol{s}_i \in \mathrm{range}(\boldsymbol{A})$, $\boldsymbol{s}_i$ is able to be linearly expressed using all columns of matrix $\boldsymbol{A}$ as:

$$\boldsymbol{s}_i = \sum_{i=1}^{k-1} \alpha_i\boldsymbol{A}_i\,, \boldsymbol{A} = [\boldsymbol{A}_1, \boldsymbol{A}_2, \dots, \boldsymbol{A}_{k-1}]. (S23)$$

We have the equation as:

$$(\boldsymbol{I}-\boldsymbol{A}\boldsymbol{A}^{\dagger})\boldsymbol{s_i} = \sum_{i=1}^{k-1} \alpha_i \boldsymbol{A}_i - \sum_{i=1}^{k-1} \alpha_i \boldsymbol{A}\boldsymbol{A}^{\dagger}\boldsymbol{A}_i \,. (S24)$$

Because of $\boldsymbol{A}\boldsymbol{A}^{\dagger}\boldsymbol{A}_i = \boldsymbol{A}_i$, the equation $(\boldsymbol{I}-\boldsymbol{A}\boldsymbol{A}^{\dagger})\boldsymbol{s_i} = \boldsymbol{0}$ is obtained.

If the parameter θ_i is none-identifiable, it shows $(\boldsymbol{I}-\boldsymbol{A}\boldsymbol{A}^{\dagger})\boldsymbol{s_i} = \boldsymbol{0}$ and $\boldsymbol{s_i} \in \mathrm{range}(\boldsymbol{A})$. □

Naturally, the parameter θ_i is identifiable if and only if $\boldsymbol{s_i}$ is decomposed as $\boldsymbol{s}_i^{(1)} \in \mathrm{range}(\boldsymbol{A})$ and $\boldsymbol{s}_i^{(2)} \in \ker(\boldsymbol{A}^T)$, and $\boldsymbol{s}_2 \neq \boldsymbol{0}$. As the basic theorem of linear algebra follows:

$$\mathrm{range}(\boldsymbol{A}) = (\ker(\boldsymbol{A}^T))^{\perp}, \dim(\mathrm{range}(\boldsymbol{A})) + \dim(\ker(\boldsymbol{A}^T)) = n, (S25)$$

we have $(\boldsymbol{I}-\boldsymbol{A}\boldsymbol{A}^{\dagger})\boldsymbol{s_i} = \boldsymbol{s}_i^{(2)} \neq \boldsymbol{0}$.

Proof of Theorem 4

Proof: The Eq. (22) is equivalent to an optimization problem as:

$$\min_{\boldsymbol{\theta}\in U(\boldsymbol{\theta}^*,\delta)} \sum_{j=1}^{M} \left\| \boldsymbol{h}\left(\boldsymbol{\varphi}\left(t_{i_j},\boldsymbol{\theta}\right)\right) - \boldsymbol{h}\left(\boldsymbol{\varphi}\left(t_{i_j},\boldsymbol{\theta}^*\right)\right) \right\|_2^2 . (S26)$$

If $\boldsymbol{\theta}$ is structurally none-identifiable, we can find a parameter $\widehat{\boldsymbol{\theta}} \in U(\boldsymbol{\theta}^*,\delta)$ that satisfies $\boldsymbol{h}\left(\boldsymbol{\varphi}(t,\widehat{\boldsymbol{\theta}})\right) = \boldsymbol{h}(\boldsymbol{\varphi}(t,\boldsymbol{\theta}^*))$, that is, $\forall \left\{t_{i_j}\right\}_{j=1}^{M} \subseteq \{t_i\}_{i=1}^{\infty}$, we have $\boldsymbol{h}\left(\boldsymbol{\varphi}\left(t_{i_j},\widehat{\boldsymbol{\theta}}\right)\right) = \boldsymbol{h}\left(\boldsymbol{\varphi}\left(t_{i_j},\boldsymbol{\theta}^*\right)\right), j = 1,2,\ldots,M$. The necessary condition of above optimization problem at the time series $\left\{t_{i_j}\right\}_{j=1}^{M}$ is written as

$$\boldsymbol{s}(\boldsymbol{\theta}^*)^T \boldsymbol{s}(\boldsymbol{\theta}^*)\left(\widehat{\boldsymbol{\theta}} - \boldsymbol{\theta}^*\right) = \boldsymbol{0}. (S27)$$

It demonstrates that $\boldsymbol{s}(\boldsymbol{\theta}^*)^T \boldsymbol{s}(\boldsymbol{\theta}^*)$ is a singular matrix, which is contradicted with the original hypothesis that $\boldsymbol{s}(\boldsymbol{\theta}^*)$ has column full rank. □

Section 2 Supplementary Figures

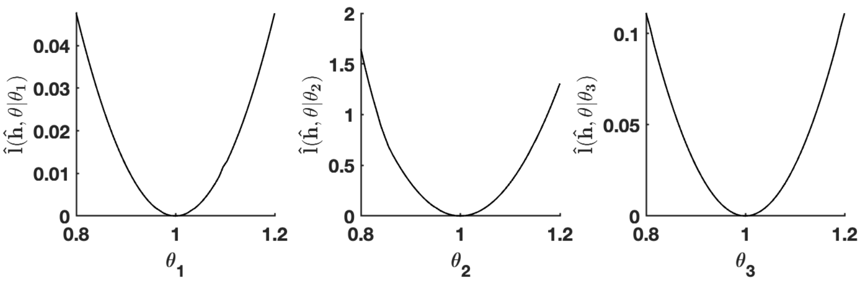

Figure S1: Practical identifiability analysis of parameters in polynomial

fitting with the loss function enhanced by parameter regularization.

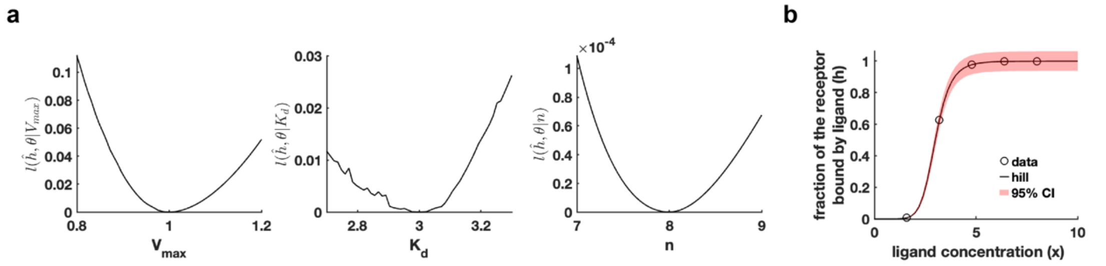

Figure S2: Practical identifiability analysis of parameters in Hill function fitting. a. Practical identifiability analysis using the profile likelihood. **b.** Uncertainty confidence interval through the perturbation to all parameters. perturbation to all parameter set as 10^{-3}.

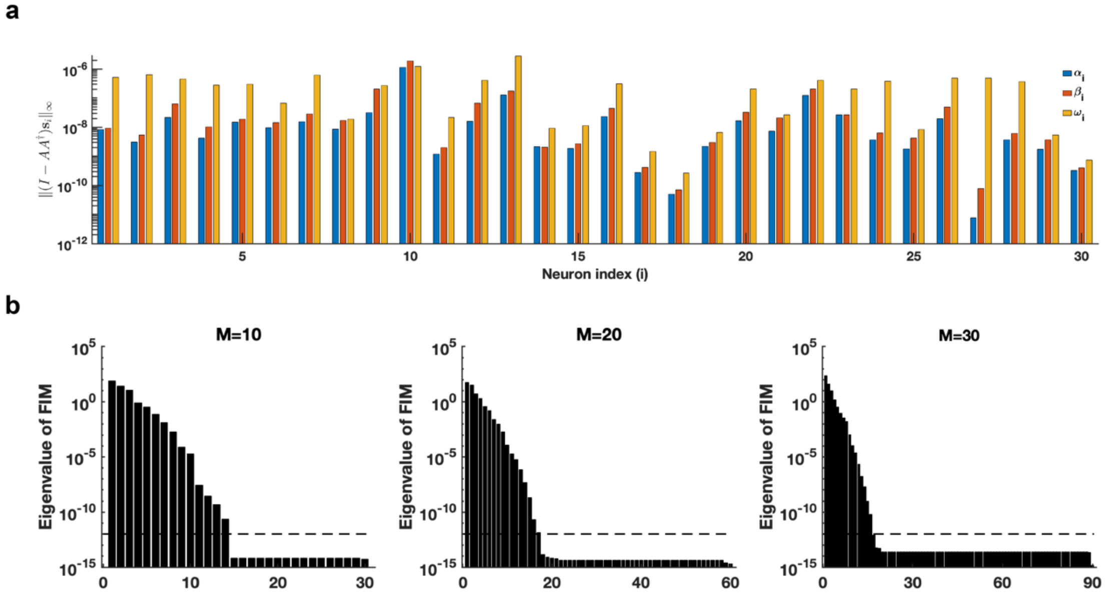

Figure S3: Practical identifiability analysis of parameters in neural network. (a). Identifiable neurons recognized by the metric $\|(I-AA^{\dagger})s_i\|_{\infty}$ when the activation function set to tanh function and the number of neurons is assigned as 30. **(b).** Eigenvalue distribution of FIM across different numbers of neurons (M). The dashed line is the threshold $\varepsilon = 10^{-12}$ of eigenvalue of $\boldsymbol{F}(\boldsymbol{\theta}^*)$. The activation function set to tanh function.

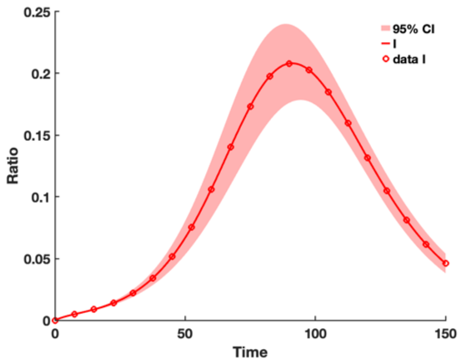

Figure S4: Practical identifiability analysis of parameters in SEIR model. Uncertainty confidence interval through the perturbation to all parameters using the synthetic data. Perturbation to all parameter set as 10^{-5}.

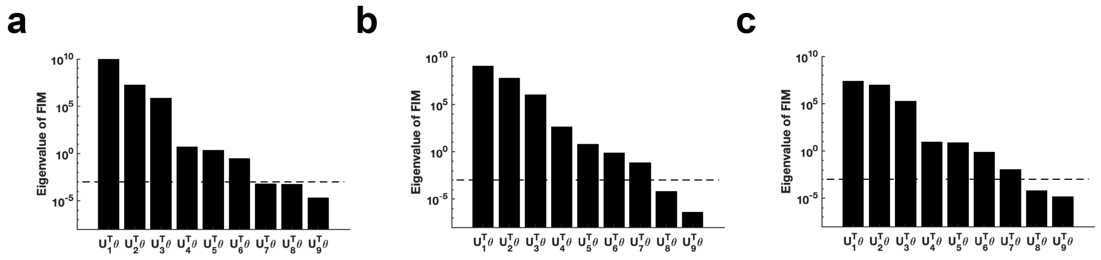

Figure S5: Practical identifiability analysis of parameters in cascade model of Alzheimer's Disease. (a) Eigenvalue of AD patients. **(b)** Eigenvalue of LMCI patients. **(c)** Eigenvalue of CN patients. The dash line is the threshold $\varepsilon = 10^{-3}$. Circles represent the real data of AD, LMCI and CN patients. The solid line represents the time course of biomarkers of patients with the given parameter values. The red area represents the 95% confidence interval under parameter perturbation.

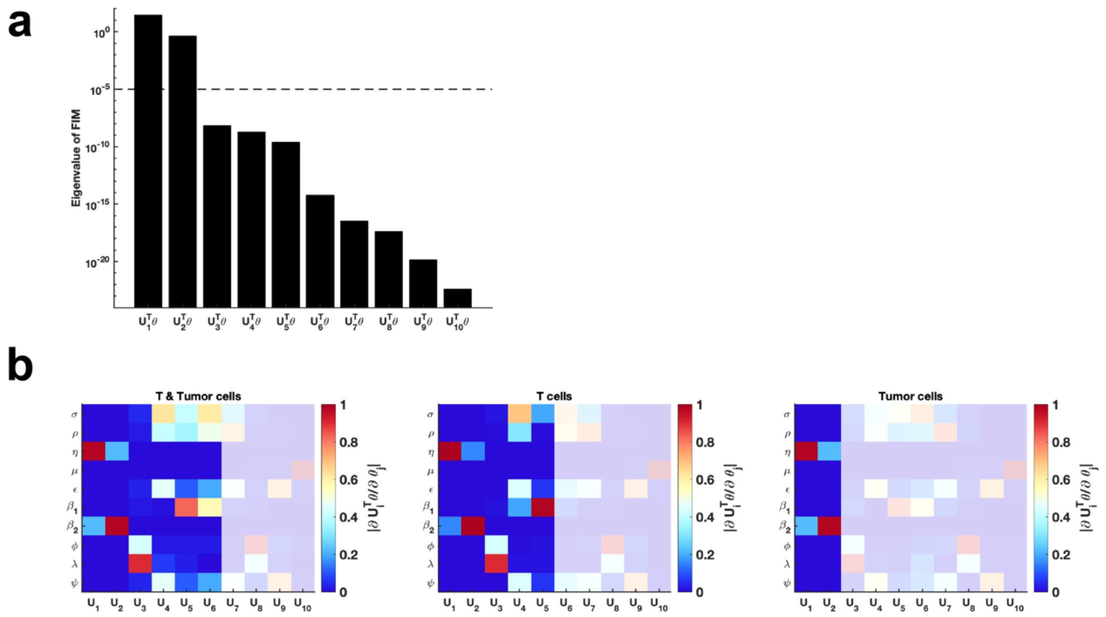

Figure S6: Practical identifiability analysis of parameters in PDE model of

cancer-immune interaction. (a). Eigenvalue of FIM using the glioblastoma data. The dash line is the threshold $\varepsilon = 10^{-5}$. **(b).** Heatmap of the eigenvector matrix in the three cases of observable variables.

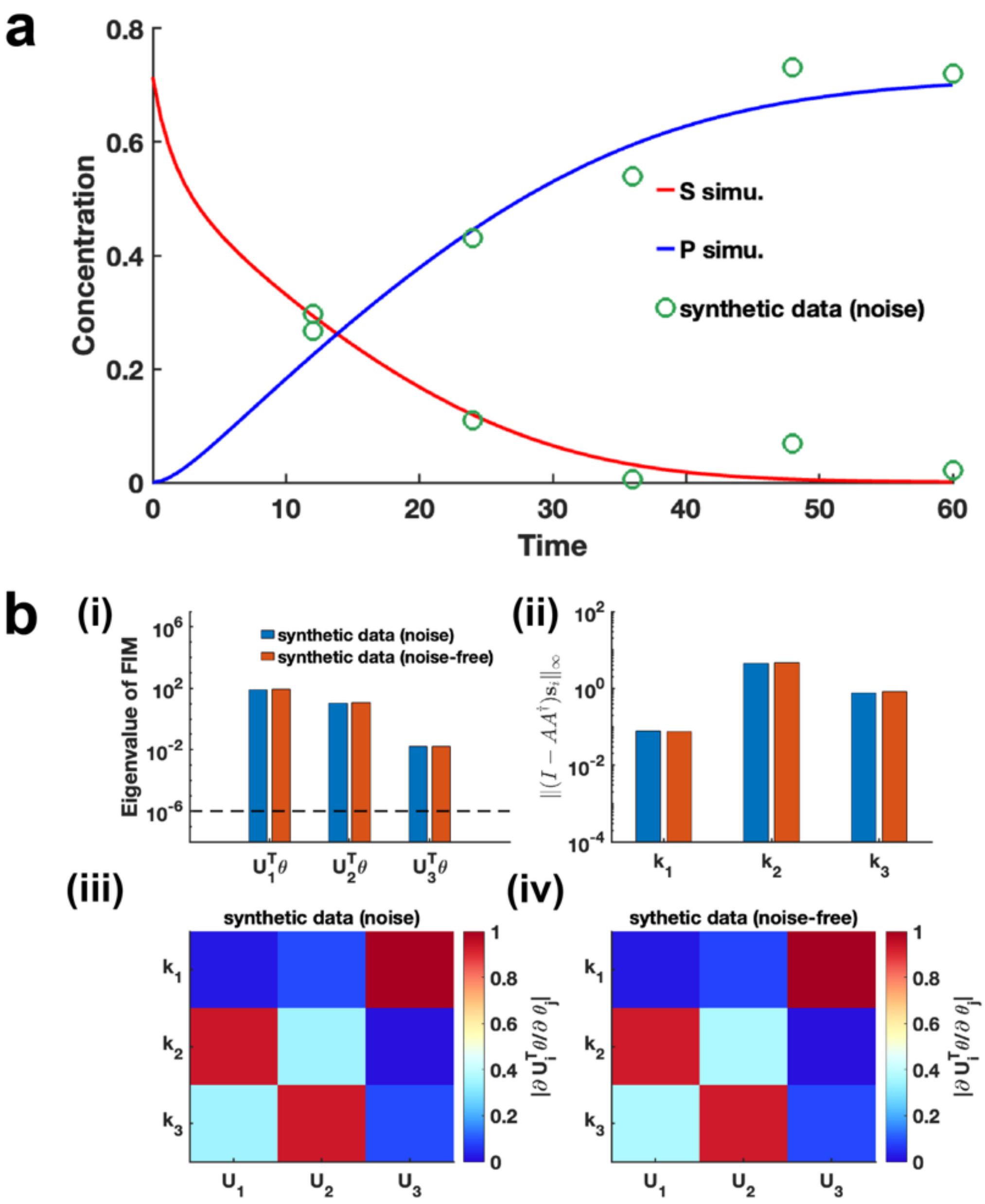

Figure S7: Practical identifiability analysis of synthetic data with additive Gaussian noise for Michaelis-Menten system. (a) Time course of substrate and product Green Circles represent the synthetic data with additive Gaussian noise drawn from a normal distribution $\mathcal{N}(0,0.05)$. (b) Parameter identifiability analysis. (i) Eigenvalue of $F(\theta^*)$ using the noise data and noise-free data. The dash line is the threshold $\varepsilon = 10^{-6}$. (ii) Coordinate identifiability analysis. (iii) Heatmap of the eigenvector matrix using synthetic data with additive Gaussian noise. (iv) Heatmap of the eigenvector matrix using noise-free synthetic data.

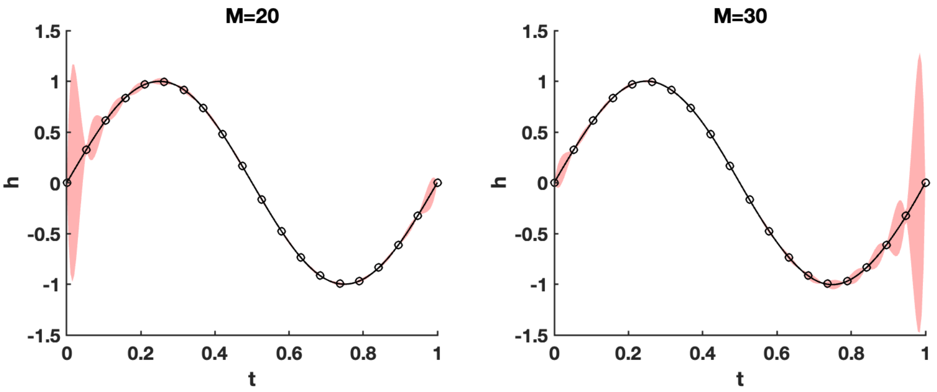

Figure S8: Uncertainty quantification was conducted by introducing perturbations to non-identifiable parameters across different numbers of neurons (M), with the activation function set to tanh and the initial parameters of the neural networks randomly initialized.

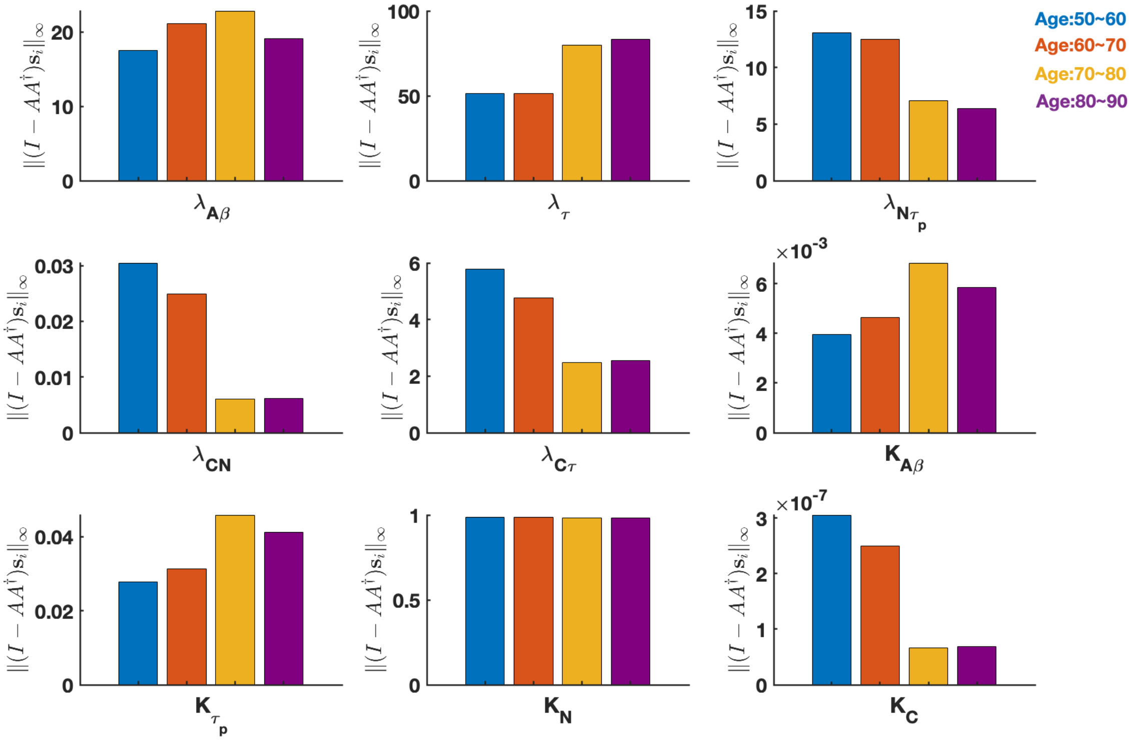

Figure S9: Coordinate identifiability analysis to parameter using the metric $\|(I - AA^{\dagger})s_i\|_\infty$ using synthetic data stratified by age.

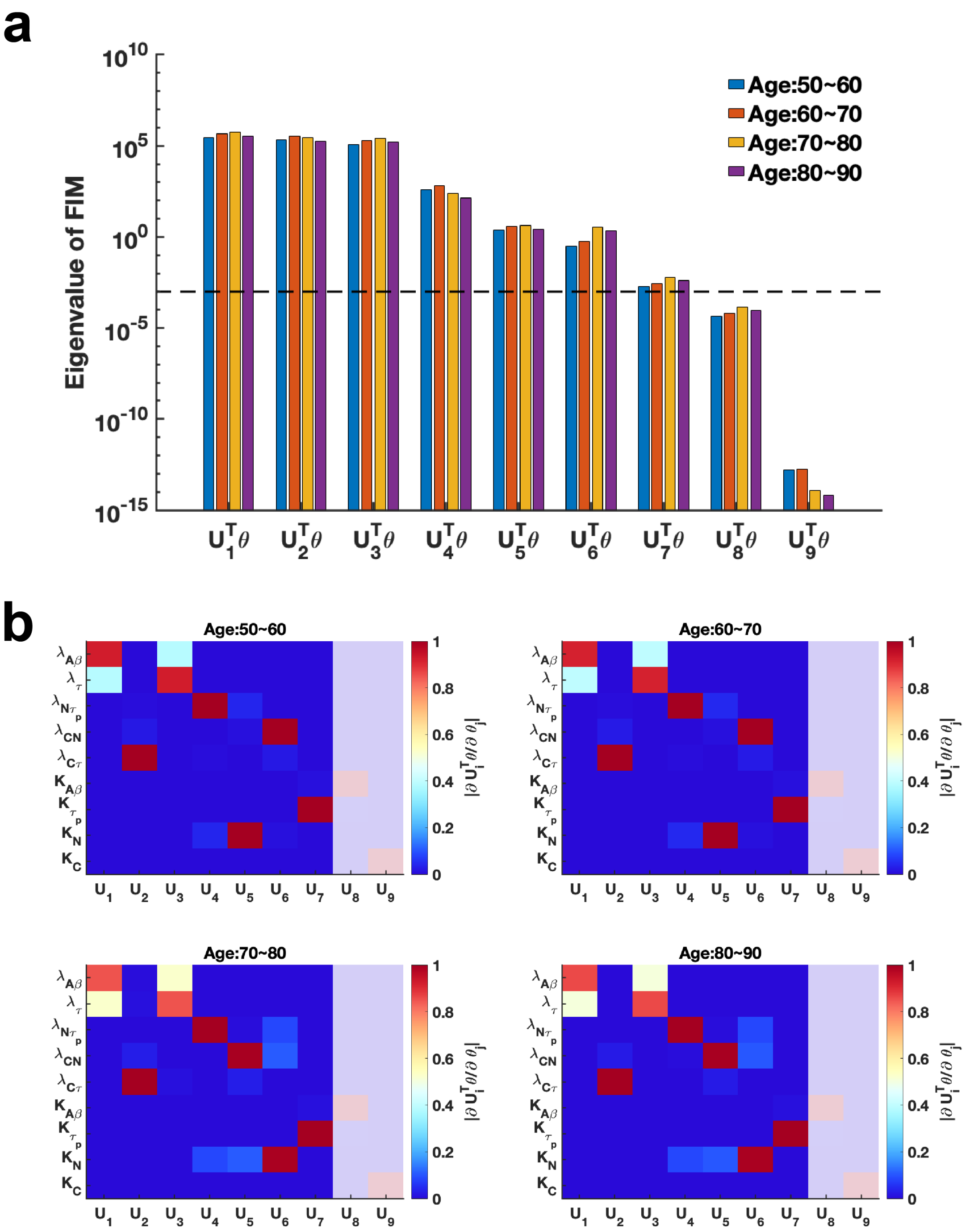

Figure S10: Practical identifiability analysis of parameters using synthetic data stratified by age in cascade model of Alzheimer's Disease. (a). Eigenvalue of FIM. The dash line is the threshold $\varepsilon = 10^{-3}$. **(b).** Heatmap of the eigenvector matrix.

Section 3 Applications of Practical Identifiability

We construct three computing examples, such as polynomial fitting, Hill function fitting, and neural network fitting, to illustrate the superiority of FIM to analyze the practical identifiability.

Polynomial fitting example. Polynomial fitting example is constructed as follows

$\varphi(t,\boldsymbol{\theta}) = \theta_1 + \theta_2 t^2 + \theta_3[(t-1)(t-2)(t-3)+2], h(\varphi) = \varphi. (S28)$

And the synthetic data is as follows:

t_j	1	2	3
$\hat{h}_j$	4	7	12

Hill function example. The formular of Hill function follows:

$$\varphi(x,\boldsymbol{\theta}) = V_{max}\frac{x^n}{x^n + K_d^n}, \boldsymbol{\theta} = [V_{max}, K_d, n], h(\varphi) = \varphi. \ (S29)$$

"The synthetic dataset is presented as follows:

x_j	1.6	3.2	4.8	6.4	8.0
$\hat{h}_j$	0.0065	0.6263	0.9772	0.9977	0.9996

Neural network example. A neural network is constructed with one hidden layer denoted as $\boldsymbol{\varphi}(t,\boldsymbol{\theta})$ to fit the function $\hat{h}(t) = \sin(2\pi t)$ $(t \in [0,1])$. The synthetic data is generated by uniformly sampling N points over the interval [0,1] denoted as $\{t_i, \hat{h}(t_i)\}_{i=1}^{N}$. The logarithmic likelihood function is the least square formular as follows:

$$l(\hat{\boldsymbol{h}};\boldsymbol{\theta}) = \frac{1}{N}\sum_{i=1}^{N}(\varphi(t_i,\boldsymbol{\theta}) - \sin(2\pi t_i))^2, t_i = \frac{i-1}{N-1};$$

$$\varphi(t,\boldsymbol{\theta}) = \sum_{j=1}^{M} w_j\sigma(\alpha_j t + \beta_j). (S30)$$

The parameter is $\boldsymbol{\theta} = [\alpha_1, \alpha_2, \ldots, \alpha_M, \beta_1, \beta_2, \ldots, \beta_M, w_1, w_2, \ldots, w_M]$, and the sensitive matrix is conducted as follows:

$\nabla\varphi(t_i,\boldsymbol{\theta}) = [\nabla\varphi(t_i,\boldsymbol{\alpha}) \quad \nabla\varphi(t_i,\boldsymbol{\beta}) \quad \nabla\varphi(t_i,\boldsymbol{\omega})]$

$$\nabla\varphi(t_i,\boldsymbol{\alpha}) = \left[w_1\frac{\partial\sigma(\alpha_1 t_i + \beta_1)}{\partial\alpha_1}, w_2\frac{\partial\sigma(\alpha_2 t_i + \beta_2)}{\partial\alpha_2}, \ldots, w_M\frac{\partial\sigma(\alpha_M t_i + \beta_M)}{\partial\alpha_M}\right],$$

$$\nabla\varphi(t_i,\boldsymbol{\beta}) = \left[w_1\frac{\partial\sigma(\alpha_1 t_i + \beta_1)}{\partial\beta_1}, w_2\frac{\partial\sigma(\alpha_2 t_i + \beta_2)}{\partial\beta_2}, \ldots, w_M\frac{\partial\sigma(\alpha_M t_i + \beta_M)}{\partial\beta_M}\right],$$

$\nabla\varphi(t_i, \boldsymbol{\omega}) = [\sigma(\alpha_1 t_i + \beta_1), \sigma(\alpha_2 t_i + \beta_2), \ldots, \sigma(\alpha_M t_i + \beta_M)]$.

We leverage two different activation functions, namely ReLU and tanh as follows:

case (1) $ReLu(x) = \begin{cases} x, & x \geq 0 \\ 0, & x < 0 \end{cases}, \frac{dReLU(x)}{dx} = \begin{cases} 1, & x \geq 0 \\ 0, & x < 0 \end{cases}$;

case (2) $\frac{d\tanh(x)}{dx} = 1 - \tanh^2(x)$

In case (1), N is chosen as 500, and M is set to 40. In case (2), N is decreased to 20 because of the smoothness of tanh.

Various biological systems. Practical identifiability analysis is widely applied to dynamic differential equation models in biological systems to assess whether model parameters can be reliably identified from available data. In this study, we evaluate the proposed parameter practical identifiability metric by leveraging it to five dynamic differential equation models: the LV model (3), the Michaelis-Menten system (4), the SEIR model (5), a cascade model of Alzheimer's disease (AD) (6), and PDE model of cancer-immune interaction model (7). A general form of ODEs is described as:

$$\frac{d\boldsymbol{\varphi}}{dt} = \boldsymbol{f}(\boldsymbol{\varphi}, \boldsymbol{\theta}),$$

$$\boldsymbol{h}(t, \boldsymbol{\theta}) = \boldsymbol{h}(\boldsymbol{\varphi}(\boldsymbol{t}, \boldsymbol{\theta})). (S31)$$

Herein, $\boldsymbol{\varphi}(\boldsymbol{t}, \boldsymbol{\theta}) \in \mathbb{R}^m$ is a vector of state variables, $\boldsymbol{h}(t, \boldsymbol{\theta}) \in \mathbb{R}^L$ is the measurement or output vector. $\boldsymbol{\theta} \in \mathbb{R}^k$ is parameter vector and assumed as constants in this work. Let $\boldsymbol{\varsigma}(t, \boldsymbol{\theta}) = \frac{\partial \boldsymbol{\varphi}}{\partial \boldsymbol{\theta}}$, it can be shown that ς satisfies

$$\frac{d\boldsymbol{\varsigma}}{dt} = \frac{\partial \boldsymbol{f}(\boldsymbol{\varphi}, \boldsymbol{\theta})}{\partial \boldsymbol{\theta}} + J(\boldsymbol{\varphi}, \boldsymbol{\theta})\boldsymbol{\varsigma}, (S32)$$

$$\boldsymbol{\varsigma}(0, \boldsymbol{\theta}) = \boldsymbol{0}.$$

Where $J(\boldsymbol{\varphi}, \boldsymbol{\theta})$ is the Jacobian $\frac{\partial \boldsymbol{f}(\boldsymbol{\varphi}, \boldsymbol{\theta})}{\partial \boldsymbol{\varphi}}$. The measurement $\boldsymbol{h}(t, \boldsymbol{\theta})$ satisfies $\frac{\boldsymbol{d}}{dt}\frac{\partial \boldsymbol{h}}{\partial \boldsymbol{\theta}} = \frac{\partial \boldsymbol{h}}{\partial \boldsymbol{\varphi}}\frac{d\boldsymbol{\varsigma}}{dt}$ so that the sensitive matrix $\boldsymbol{s}(\boldsymbol{\theta})$ is obtained by Eqs. (S31-S32) as follows:

$$\frac{d\boldsymbol{\varphi}}{dt} = \boldsymbol{f}(\boldsymbol{\varphi}, \boldsymbol{\theta}),$$

$$\frac{d\boldsymbol{\varsigma}}{dt} = \frac{\partial \boldsymbol{f}(\boldsymbol{\varphi}, \boldsymbol{\theta})}{\partial \boldsymbol{\theta}} + J(\boldsymbol{\varphi}, \boldsymbol{\theta})\boldsymbol{\varsigma},$$

$$\frac{\boldsymbol{d}}{dt}\frac{\partial \boldsymbol{h}}{\partial \boldsymbol{\theta}} = \frac{\partial \boldsymbol{h}}{\partial \boldsymbol{\varphi}}\frac{d\boldsymbol{\varsigma}}{dt}, (31)$$

$$\boldsymbol{h}(t, \boldsymbol{\theta}) = \boldsymbol{h}(\boldsymbol{\varphi}(\boldsymbol{t}, \boldsymbol{\theta})),$$

$$\boldsymbol{\varsigma}(0, \boldsymbol{\theta}) = \boldsymbol{0}, \boldsymbol{\varphi}(0, \boldsymbol{\theta}) = \boldsymbol{\varphi}_0.$$

LV model. The classical LV model (3) describes the dynamics of prey and predator as follows:

$$\frac{dx}{dt} = \alpha x - \beta xy,$$

$$\frac{dy}{dt} = \delta xy - \gamma y. \ (S33)$$

Herein, the parameter is $\boldsymbol{\theta} = [\alpha, \beta, \delta, \gamma]^{\boldsymbol{T}}$ and the observable variable is $\boldsymbol{h}(t, \boldsymbol{\theta}) = [x(t, \boldsymbol{\theta}), y(t, \boldsymbol{\theta})]$. The variable x and y represent the prey and predator, respectively.

Michaelis–Menten system. The Michaelis-Menten system (8) is used to model the enzyme reaction or ligand-receptor response as follows:

$$\frac{d[S]}{dt} = -k_1[S][E] + k_2[ES],$$

$$\frac{d[E]}{dt} = -k_1[S][E] + (k_2 + k_3)[ES],$$

$$\frac{d[ES]}{dt} = k_1[S][E] - (k_2 + k_3)[ES],$$

$$\frac{d[P]}{dt} = k_3[ES]. \ (S34)$$

Herein, $\boldsymbol{\theta} = [k_1, k_2, k_3]^{\boldsymbol{T}}$. We consider two cases of observable variable as follows:

case (1) $\boldsymbol{h}(t, \boldsymbol{\theta}) = [y_1(t, \boldsymbol{\theta}), y_4(t, \boldsymbol{\theta})]$;

case (2) $\boldsymbol{h}(t, \boldsymbol{\theta}) = y_4(t, \boldsymbol{\theta})$.

SEIR model. The SEIR model is the classical compartmental model to understand the disease dynamics (5) as follows:

$$\frac{dS}{dt} = -\beta SI,$$

$$\frac{dE}{dt} = \beta SI - \sigma E,$$

$$\frac{dI}{dt} = \sigma E - \gamma I,$$

$$\frac{dR}{dt} = \gamma I. (S35)$$

Herein $\boldsymbol{\theta} = [\beta, \sigma, \gamma]^T$. There are four cases of observable as follows:

case (1) $\boldsymbol{h}(t, \boldsymbol{\theta}) = I(t, \boldsymbol{\theta})$;

case (2) $\boldsymbol{h}(t, \boldsymbol{\theta}) = [E(t, \boldsymbol{\theta}), I(t, \boldsymbol{\theta})]$;

case (3) $\boldsymbol{h}(t, \boldsymbol{\theta}) = [S(t, \boldsymbol{\theta}), I(t, \boldsymbol{\theta})]$;

case (4) $\boldsymbol{h}(t, \boldsymbol{\theta}) = [S(t, \boldsymbol{\theta}), E(t, \boldsymbol{\theta}), I(t, \boldsymbol{\theta})]$.

Cascade model of Alzheimer's Disease. Our group previously developed the cascade model of Alzheimer's Disease (6) as follows:

$$\frac{dA_\beta}{dt} = \lambda_{A_\beta} A_\beta \left(1 - \frac{A_\beta}{K_{A_\beta}}\right),$$

$$\frac{d\tau_p}{dt} = \lambda_\tau A_\beta \left(1 - \frac{\tau_p}{K_{\tau_p}}\right),$$

$$\frac{dN}{dt} = \lambda_{N_{\tau_p}} \tau_p \left(1 - \frac{N}{K_N}\right),$$

$$\frac{dC}{dt} = \left(\lambda_{CN} N + \lambda_{C_\tau} \tau_p\right)\left(1 - \frac{C}{K_C}\right). \quad (S36)$$

Where the observable variable is $\boldsymbol{h}(t,\boldsymbol{\theta}) = \left[A_\beta(t,\boldsymbol{\theta}), \tau_p(t,\boldsymbol{\theta}), N(t,\boldsymbol{\theta}), C(t,\boldsymbol{\theta})\right]$, and the parameter is $\boldsymbol{\theta} = \left[\lambda_{A_\beta}, \lambda_\tau, \lambda_{N_{\tau_p}}, \lambda_{CN}, \lambda_{C_\tau}, K_{A_\beta}, K_{\tau_p}, K_N, K_C\right]^{\boldsymbol{T}}$.

PDE model of cancer-immune interactions: The model of tumor-immune interactions consists of three dependent variables denoted E, T and C, which are the local densities/concentrations of tumor-infiltrating cytotoxic lymphocytes (TICLs), tumor cells, TICL–tumor cell complexes, respectively. The formula of nondimensional model (7) follows as:

$$\frac{\partial E}{\partial \bar{t}} = \nabla^2 E + \sigma\chi(x) + \frac{\rho C}{\eta + T} - \sigma E - \mu E T + \epsilon C,$$

$$\frac{\partial T}{\partial \bar{t}} = \omega\nabla^2 T + \beta_1 T(1 - \beta_2 T) - \phi E T + \lambda C,$$

$$\frac{\partial C}{\partial \bar{t}} = \mu E T - \psi C. \, (S37)$$

$$\chi(x) = \begin{cases} 0, & \text{if } x \le l = 0.2 \\ 1, & \text{if } x > l = 0.2 \end{cases}, x \in [0,1]$$

The initial conditions are given by

$$E(x) = \begin{cases} 0, & x \le l \\ (1 - \exp(-1000(x-l)^2)), & x > l \end{cases}$$

$$T(x) = \begin{cases} T_0(1 - \exp(-1000(x-l)^2)), & x \le l \\ 0, & x > l \end{cases}$$

$$C(x) = C_0 \exp(-1000(x-l)^2)$$

We use the non-flux boundary conditions as follows:

$$\frac{\partial E}{\partial x}(0,t) = \frac{\partial E}{\partial x}(1,t) = \frac{\partial T}{\partial x}(0,t) = \frac{\partial T}{\partial x}(1,t) = \frac{\partial C}{\partial x}(0,t) = \frac{\partial C}{\partial x}(1,t) = 0$$

The parameter is $\boldsymbol{\theta} = [\sigma, \rho, \eta, \mu, \epsilon, \beta_1, \beta_2, \phi, \lambda, \mu, \psi]^{\boldsymbol{T}}$ and there are four observable variables we consider as follows:

case (1) $\boldsymbol{h}(t,\boldsymbol{\theta}) = [\int_0^1 E(t,x;\boldsymbol{\theta})dx, \int_0^1 T(t,x;\boldsymbol{\theta})dx\,]$

case (2) $\boldsymbol{h}(t,\boldsymbol{\theta}) = E(t,\boldsymbol{x};\boldsymbol{\theta})$,

$$E(t,\boldsymbol{x};\boldsymbol{\theta}) = [E(t,x_0;\boldsymbol{\theta}), E(t,x_2;\boldsymbol{\theta}), \ldots, E(t,x_N;\boldsymbol{\theta})], x_i = \tfrac{i}{N}, i = 0,1,2,\ldots,N$$

case (3) $\boldsymbol{h}(t,\boldsymbol{\theta}) = T(t,\boldsymbol{x};\boldsymbol{\theta})$,

$$T(t,\boldsymbol{x};\boldsymbol{\theta}) = [T(t,x_0;\boldsymbol{\theta}), T(t,x_2;\boldsymbol{\theta}), \ldots, T(t,x_N;\boldsymbol{\theta})],\; x_i = \tfrac{i}{N}, i = 0,1,2,\ldots,N$$

case (4) $\boldsymbol{h}(t,\boldsymbol{\theta}) = [E(t,\boldsymbol{x};\boldsymbol{\theta}), T(t,\boldsymbol{x};\boldsymbol{\theta})]$,

$E(t,\boldsymbol{x};\boldsymbol{\theta}) = [E(t,x_0;\boldsymbol{\theta}), E(t,x_2;\boldsymbol{\theta}), \ldots, E(t,x_N;\boldsymbol{\theta})]$,

$T(t,\boldsymbol{x};\boldsymbol{\theta}) = [T(t,x_0;\boldsymbol{\theta}), T(t,x_2;\boldsymbol{\theta}), \ldots, T(t,x_N;\boldsymbol{\theta})]$, $x_i = \frac{i}{N}, i = 0,1,2,\ldots,N$

Section 4 Values of the parameters

Figure 2. Perturbation to non-identifiable parameter set as 10, and perturbation to all parameter set as 0.1.

Figure 3. Perturbation to non-identifiable parameter set as 10. The threshold of eigenvalue for critical data (ε) is set to 1×10^{-5}.

Figure 4. Perturbation to non-identifiable parameter set as 10^{11}.

Figure 5. $\boldsymbol{\theta}^* = [0.545,0.028,0.024,0.803]^T$; Perturbation to all parameter set as 10^{-6}; $\boldsymbol{\varphi_0} = [33.956,5.933]^T$ (9).

Figure 6. $\boldsymbol{\theta}^* = [10^6, 10^{-4}, 0.1]^T$ (8); Perturbation to non-identifiable parameter set as 0.5; $\boldsymbol{\varphi_0} = [5 \times 10^{-7}, 2 \times 10^{-7}, 0,0]^T$ (8). The threshold of eigenvalue for critical data (ε) is set to 5×10^{-5}.

Figure 7. Synthetic data: $\boldsymbol{\theta}^* = [0.2,0.1,0.06]^T$, $\boldsymbol{\varphi_0} = [0.99,0.01,0,0]^T$ and Perturbation to non-identifiable parameter set as 10^{-2}.
Influenza data: $\boldsymbol{\theta}^* = [5.4, 5.8, 4.54]^T$, $\boldsymbol{\varphi_0} = [1.0,0,1.9 \times 10^{-5}, 0]^T$ and Perturbation to non-identifiable parameter set as 100.

Figure 8. AD data:
$\boldsymbol{\varphi_0} = [36.03,12,38,0.18,1.68]^T$ (6),
$\boldsymbol{\theta}^* = [0.349,0.0182,0.058,0.165,0.041,259.446,123.350,0.9997,50,4803]^T$,
Perturbation to non-identifiable parameter set as 10^{-4};
LMCI data:
$\boldsymbol{\varphi_0} = [36.03,12,38,0.22,1.68]^T$ (6)
$\boldsymbol{\theta}^* = [0.237,0.0066,0.01,0.167,0.021,259.440,123.350,1.00,50,480]^T$,
Perturbation to non-identifiable parameter set as 10^{-4};
CN data:
$\boldsymbol{\varphi_0} = [44.92,3.68,0.52,1.68]^T$ (6),
$\boldsymbol{\theta}^* = [0.1136,0.0096,0.0196,0.0147,0.077,139.94,123.350,1.00,50,480]^T$,
Perturbation to non-identifiable parameter set as 10^{-4}.
Age synthetic data:
$\boldsymbol{\varphi_0} = [36.03,12.38,0.18,1.68]^T$,
$\boldsymbol{\theta}^* = [0.0652,0.15,0.02,0.0167,0.0000083,139.94,123.350,1.00,50,480]^T$,

Figure 9. Glioblastoma data:
$\boldsymbol{\theta}^* = [6.86 \times 10^4, 7.49 \times 10^6, 2.74, 2.6 \times 10^6, 3.12 \times 10^7, 1.8 \times 10^5, 0.808, 2.57 \times 10^5, 2.37 \times 10^6, 3.12 \times 10^7]^T$,
$\boldsymbol{\varphi_0} = [1.92 \times 10^6, 2.0 \times 10^7, 1.92 \times 10^6]^T$ (7)
Perturbation to non-identifiable parameter set as 5×10^8 . The spatial discretization number N is assigned as 128 in the one-dimensional space.
Synthetic data:
$\boldsymbol{\theta}^* = [4.12 \times 10^4, 59960, 0.0404, 6.5 \times 10^7, 3.12 \times 10^7, 1.8 \times 10^5, 1.0, 4.29 \times 10^5, 1.58 \times 10^4, 3.12 \times 10^7]^T$,
Perturbation to non-identifiable parameter set as 3×10^8 . The spatial discretization number N is assigned as 128 in the one-dimensional space.

Section 5 Supplementary Tables

Table S1 Parameter of Cascade model of Alzheimer's Disease (6)

Parameter	Biological description
λ_{A_β}	Growth rate of amyloid beta
λ_τ	Growth rate of Tau protein
$\lambda_{N_{\tau_p}}$	Growth rate of Neurodegeneration associated with Tau protein
λ_{CN}	Growth of cognitive decline associated with neurodegeneration
λ_{C_τ}	Growth of cognitive decline associated with Tau protein
K_{A_β}	carrying capacity of amyloid beta
K_{τ_p}	carrying capacity of Tau protein
K_N	carrying capacity of Neurodegeneration
K_C	carrying capacity of cognitive decline

Table S2 Parameter of PDE model of cancer-immune interaction (7)

Parameter	Biological description
σ	Normal rate of flow of mature lymphocytes into the tissue
ρ	Proliferation rate of T cells
η	EC50 of tumor cells
μ	positive constant of 'local' kinetic
ϵ	positive constant of 'local' kinetic
β_1	maximal growth rate of the tumor cell
β_2	maximal growth rate of the tumor cell
ϕ	positive constant of 'local' kinetic
λ	positive constant of 'local' kinetic
μ	positive constant of 'local' kinetic
ψ	positive constant of 'local' kinetic
ω	random motility coefficient of the tumor cells

Table S3 Threshold values chosen in different cases

Case	Threshold value (ε)
Polynomial Fitting	10^{-4}
Hill Function	10^{-4}
Neural Network	10^{-12}
LV Model	10^{-6}
Michaelis-Menten System	10^{-6}
SEIR Model	10^{-6}
Cascade Model of Alzheimer's Disease	10^{-3}
PDE Model of Cancer-Immune System	10^{-5}

References

1. T. J. Rothenberg, Identification in parametric models. *Econometrica: Journal of the Econometric Society* 577–591 (1971).

2. C. Kreutz, An easy and efficient approach for testing identifiability. *Bioinformatics* **34**, 1913–1921 (2018).

3. J. D. Murray, *Mathematical biology*, 3rd ed (Springer, 2002).

4. J. Lei, *Systems Biology* (Springer International Publishing, 2021).

5. E. Hunter, J. D. Kelleher, Understanding the assumptions of an SEIR compartmental model using agentization and a complexity hierarchy. *Journal of Computational Mathematics and Data Science* **4**, 100056 (2022).

6. W. Hao, S. Lenhart, J. R. Petrella, Optimal anti-amyloid-beta therapy for Alzheimer's disease via a personalized mathematical model. *PLoS Comput Biol* **18**, e1010481 (2022).

7. A. Matzavinos, M. A. J. Chaplain, V. A. Kuznetsov, Mathematical modelling of the spatio-temporal response of cytotoxic T-lymphocytes to a solid tumour. *Mathematical Medicine and Biology* **21**, 1–34 (2004).

8. D. J. Higham, Modeling and simulating chemical reactions. *SIAM Review* **50**, 347–368 (2008).

9. P. Howard, Modeling basics. *Lecture Notes for Math* **442** (2009).
